# Instinct, Evolution, and Dreams

**Published:** 1905-11-11

**Authors:** 


### Instinct, Evolution, and Dreams.

What is Instinct? The most concise interpretation of the term is that which defines instinct as the experience of the race in contrast to reason which is the experience of the individual. "Instinct" is really a psychological concept whose innate nature is not well understood, but for whose study a wide and profitable field is open. The term "instinct" was long regarded as a metaphysical-theological conception, and it was not until Maudsley showed that many problems of the mind are dependent on the ordinary laws of physiology that the science of medico-psychology was established. Professor Loeb, in his "Studies in General Physiology," asserts that what have been taken for the voluntary or instinctive movements in animals are in reality the effects of light, of gravity, of friction, of chemical forces, and other external influences, and he gives examples which appear favourable to his statement. According to Loeb, the true problem is the analysis of the circumstances which in each case determine unequivocally the reflex movements of an animal, and these ought to be investigated in the same way as physical or chemical phenomena.

But we know that true instincts exist; we know that they have a practical as well as a theoretical value; but, so far, we have not been able to analyse them accurately. If they are regarded as manifestations of subconscious hereditary memory, there is at least a good base from which to start a scientific inquiry. Hughlings Jackson has proved the existence of different nerve levels. The highest is the moral sense which may very occasionally be disturbed without involving the reasoning and emotional faculties; these are the controlling agents in civilised man, and did not exist in our prehistoric ancestors, who did not hesitate when hungry to seize any savoury morsel, or, when angry, to retaliate regardless of the consequences. The milder grades of mental disorder, by putting out of action the higher controlling faculties, reduce the patient to the mental level of such ancestors. A further mental reduction eliminates the reasoning faculty, and the patient becomes a mere creature of instinct. At a still lower level only the mere animal functions remain.

In connection with the term hereditary memory which we have used above, is is of much interest to consider the many seemingly inexplicable phenomena exhibited in dreams and allied brain-states. In a recent article in the "Literary Guide" Marsh Beadnell attempts to explain some of the conditions of mind which at times occur to every one during sleep. He takes first the "falling through space" dream, and points out that after suffering the mental agony of "falling," the sleeper escapes the shock of the actual "stopping." His explanation is that the "falling" sensations have been transmitted from our remote ancestors who were fortunate enough to save themselves, after falling from great heights (tree tops), by clutching the branches. The molecular changes in the cerebral cells due to the shock of "stopping" could not be transmitted, because victims falling to the bottom would either be killed outright or die from secondary causes before being able to reproduce their kind. In a similar manner, by reverting to the habits of animals which existed centuries ago, Beadnell finds an explanation for the mental states experienced by individuals in various dreams—the pursuing monster dream, the reptile and vermin dream, colour dreams, suffocation dreams, dream passions, flying dreams.

It is indeed highly probable that there exists a connection between instincts so-called and some of those complex conditions of the mind experienced in dreams and like mental states. The problem is not merely one for peaceful meditation. An active investigation carried out on scientific lines with a view to throwing light upon the nature and origin of instinct would almost certainly yield valuable and instructive information.

